# Ultrasound in the diagnosis and differential diagnosis of enoral and plunging ranula: a detailed and comparative analysis

**DOI:** 10.1007/s40477-022-00743-7

**Published:** 2022-12-17

**Authors:** Michael Koch, Konstantinos Mantsopoulos, Victoria Leibl, Sarina Müller, Heinrich Iro, Matti Sievert

**Affiliations:** Department of Otorhinolaryngology and Head and Neck Surgery, University of Erlangen–Nuremberg, Waldstrasse 1, 91054 Erlangen, Germany

**Keywords:** Ranula, Enoral, Plunging, Diagnosis, Differential diagnosis, Ultrasound

## Abstract

**Purpose:**

To develop sonographic criteria for ranula that to allow rapid and precise diagnosis, differentiation between enoral (ER) and plunging ranula (PR), and differential diagnosis from other competing pathologies in this region.

**Methods:**

Patients who presented with or were referred with ranula between 2002 and 2022 were assessed in a retrospective study. After clinical investigation, ultrasound examinations were performed in all cases. Several sonographic parameters describing the echotexture, shape and size of ranulas, their relationship to important surrounding anatomical landmarks and the characteristic spreading pattern of ERs and PRs were elaborated and evaluated.

**Results:**

207 ranulas were included (82.12% ERs and 17.87% PRs). The ranulas were all in close anatomical relationship to the sublingual gland (SLG) and mylohyoid muscle (MM). The echo texture was hypoechoic to anechoic in 97.6% of the lesions. In comparison with ERs, PRs were larger and irregular in shape significantly more often (*P* = 0.0001). There were significant differences between ERs and PRs in their exact location relative to the SLG (superficial, deep, anterior, each *P* = 0.0001; posterior, *P* = 0.03) and level of the MM (above, below, above and below, *P* = 0.0001 each). The exact extent and plunging pattern were depicted in all PRs, but naturally in none of the ERs.

**Conclusions:**

The ultrasound criteria developed in this study, confirming previously published results, indicate that ultrasound is an excellent diagnostic tool for diagnosing ranula and differentiating between ERs and PRs.

## Introduction

Ranula is a rare pathological condition that originates in the sublingual gland (SLG) [1]. In the majority of cases, it develops after inflammation and/or mechanical trauma leading to obstruction of the excretory Rivinus duct (minor sublingual duct) of a vertically oriented minor gland, or also of the Bartholin duct of the greater gland [1–4], and an enoral ranula (ER) or a more complicated plunging ranula (PR) may occur. PRs are mainly located below the level of the mylohyoid muscle (MM) and can extend through a hiatus of the MM or can develop around the posterior border of the MM into the submandibular space (SS) or parapharyngeal space (PS) [1, 3, 5, 6].

The diagnosis of ranula can usually be established quickly and easily by clinical investigation, but differentiation between ER and PR may be difficult. Imaging techniques like ultrasound, MRI and CT-scan supplement this by assessing the exact extent of ranulas [1, 6–13]. The differential diagnosis (DD) includes salivary gland cysts, vascular malformations, various other cystic lesions located in the floor of the mouth, the SS or PS, and the anterior cervical or thyroid region [1, 10, 12, 14–16].

Ultrasound has been described as a useful diagnostic tool [6, 11, 17], in particular in prenatal or pediatric ranulas [18]. In ER transcervical^19^, but also transoral US [20], have proved to be promising diagnostic tools. The size, precise location, and extent of ERs or PRs can be determined using ultrasound. In PRs, specific aspects such as extension through a hiatus of the MM, or development of ranula in the posterior space over the MM and then around its posterior border into the SS, have been investigated in several publications [6, 11, 12, 17]. Not surprisingly, some authors have suggested that ultrasound should be used as a first-line diagnostic tool, particularly in children and young patients [1, 11, 18].

The aim of this retrospective study was to identify detailed ultrasound parameters for ultrasound-based diagnosis of ER and PR in order to allow rapid and precise diagnosis, differentiation between ER and PR, and differential diagnosis from other competing pathologies in this region.

## Patients and methods

This retrospective study was carried out in the Department of Otorhinolaryngology, Head and Neck Surgery at the Friedrich–Alexander University of Erlangen-Nuremberg.

Patients who presented in our department between January 2002 and April 2022 due to suspected ranula were included. The patients were investigated by clinical examination and ultrasonography. If indicated, additional diagnostic measures such as ultrasound-guided puncture or MRI were also used. The ultrasound examination was carried out or supervised by certified otolaryngologists with several years’ experience using high-end ultrasound devices (Siemens Sonoline Elegra in 2005–2011, ACUSON S2000 and S3000 in 2012–2019, and ACUSON Siemens Sequoia from 2019 to 2022; Siemens Medical Solutions USA Inc., Malvern, Pennsylvania, USA) and linear transducers at 4–9 MHz (9L4) or 4–10 MHz (10L4).

The patients’ age, gender, and total number of ERs or PRs were noted.

The following parameters were calculated or measured for ER and/or PR and included in the evaluation: side, laterality (unilateral/bilateral), maximum diameter, number of ranulas per case, echogenicity (markedly hypoechoic to anechoic versus moderately hypoechoic), delineation/shape (oval/round/regular versus irregular), location in relationship to the MM (partly over the MM, completely over the MM, partly below the MM, completely below the MM, partly over and below the MM), location in the SS or PS, location relative to the SLG (deep, superficial, anterior, posterior), pattern of plunging in PRs (through a hiatus of the MM, beyond the posterior border of the MM), prolapse of the SLG through a hiatus of the MM, perfusion (yes versus no). Any use of additional diagnostic measures such as puncture of the ER/PR was also noted, as well as any indication for MRI.

The aim was to identify parameters that can be used to establish a diagnosis of ER or PR. Cases were grouped according to the presence of ER or PR. It was clear that if statistical analysis were to be performed, then for significant differences between ERs and PRs to be shown, bias would be inevitable for parameters that are dependent on the diagnostic definition. Independent parameters were size, shape, and location relative to the SLG. Nevertheless, the results were helpful for illustrating differences between ERs and PRs on the basis of ultrasound findings.

### Statistical analysis

The software program SPSS Statistics, version 24, was used (IBM Corporation, Armonk, New York, USA). All data are given as means ± SEM, range, and median. Differences between groups were calculated using the Mann–Whitney *U-*test *(*exact-test) for ordinal variables, and differences between categorical variables using the chi-squared-test (exact-test). The significance level was set at *P* ≤ 0.05.

## Results

A total of 205 patients were included. Their mean age was 33.74 years, and 107 were male (52.2%). A total of 207 ranulas were investigated, 114 on the left (55.1%) and 93 on the right side (44.9%). 170 ERs (82.12%) and 37 PRs (17.87%) were diagnosed.

The correct diagnosis was confirmed by clinical and ultrasound examinations alone in all but five cases (2.44%). In the latter cases (two with perfusion, three with location in the SS only; 13.5% of all PRs), puncture of ranulas and MRI was performed to rule out an (accompanying) vascular malformation.

**Fig. 1:**
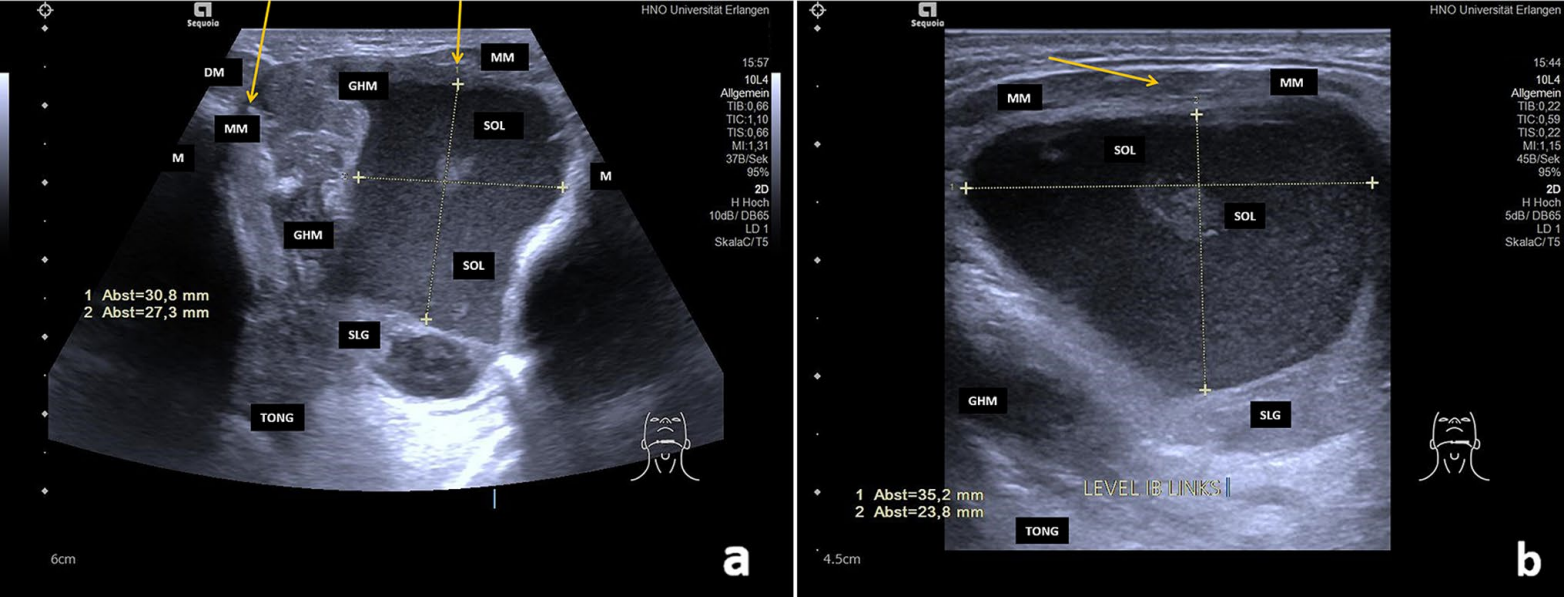
**a**–**b** Anterior **a** and lateral transection view **b** view of the floor of the mouth shows an ER in a 11-year old female patient (size 31 × 27x35mm): the ER (SOL) is located anterior, superficial and deep to the SLG and above the intact MM (orange arrows) in the floor of the mouth. The internal structure is characterized by a moderate hypoechoic tissue pattern, the echogenicity is sludge-like due to a thickened mucous secretion. *GHM* geniohyoid muscle, *DM* digastric muscle, *MM* mylohyoid muscle, *SOL* space occupying lesion/ER, *SLG* sublingual gland, *TONG* tongue, *M* mandibula, *Level IB links* Level IB left side

**Fig. 2:**
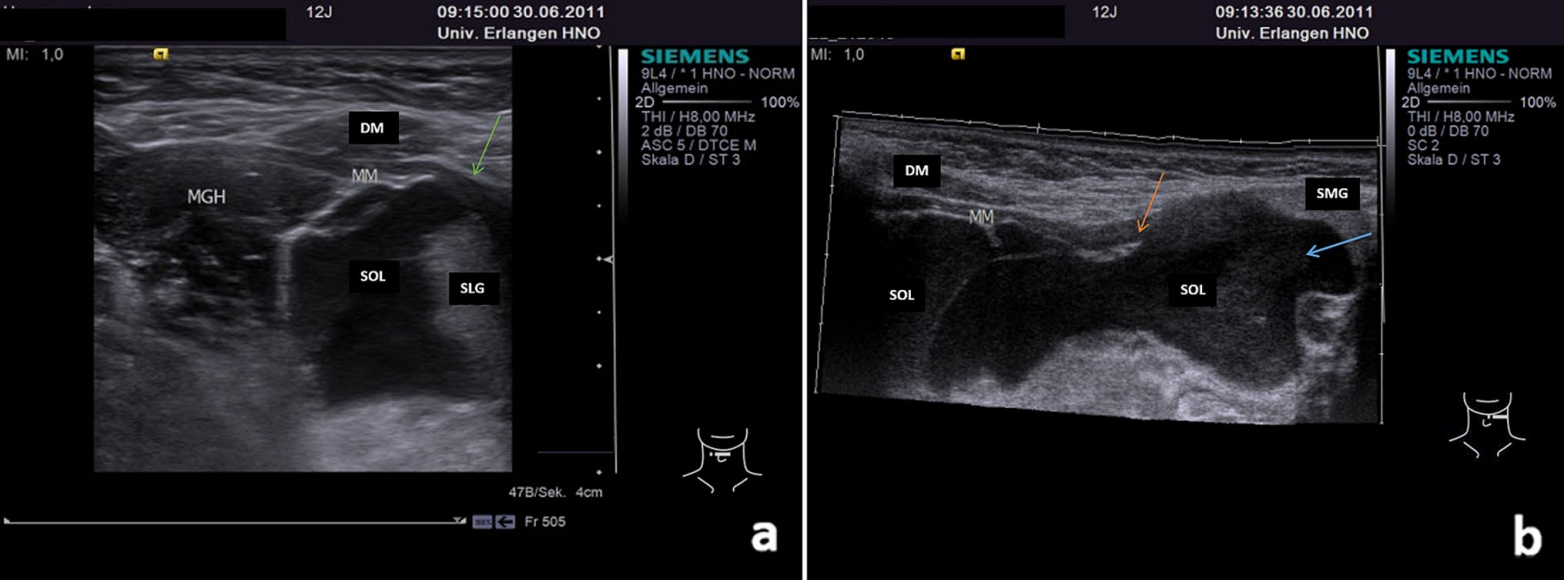
**a**–**b** Anterior (**a**) and lateral transection view (**b**) of the floor of the mouth showing a PR with a hypoechoic tissue pattern with an irregular shape in a 12 year old female patient (size 33 × 31 × 55 mm): the PR (SOL) is located anterior, superficial and deep to the SLG and above the intact MM (**a**, green arrow) in the (anterior part of the) floor of the mouth. The PR is also posteriorly located above the MM and is extending beyond the posterior border of the MM (**a**, orange arrow). The huge plunging part of the ranula, extending into the submandibular space, is indicated (**b**, blue arrow). *MGH* geniohyoid muscle, *DM* digastric muscle, *MM* mylohyoid muscle, *SOL* space occupying lesion/PR, *SLG* sublingual gland, *SMG* submandibular gland

**Fig. 3:**
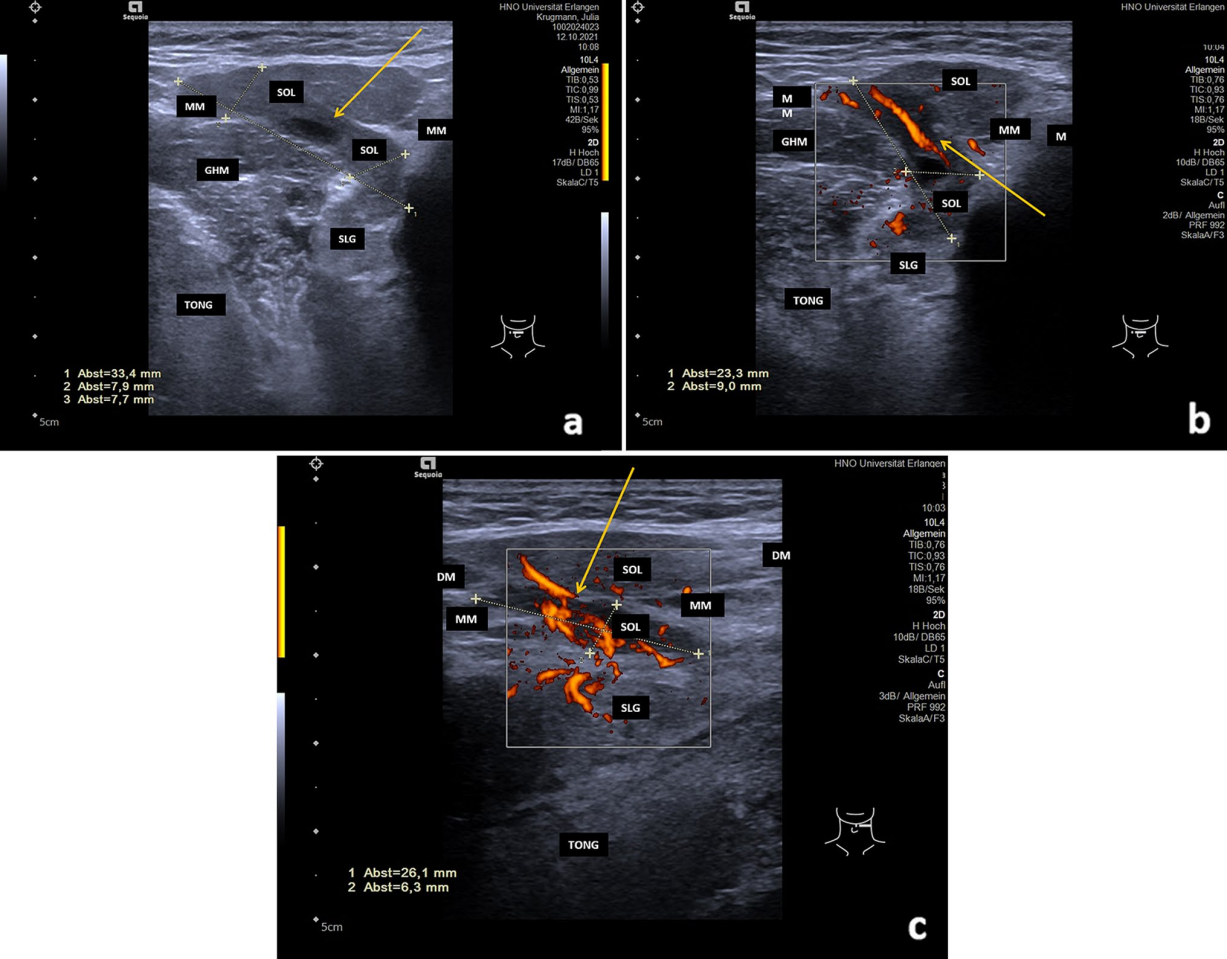
**a**–**c** Anterior transection view of the floor of the mouth native (**a**) and with low-flow doppler sonography (**b**) shows a PR in a 23 year old female patient (size 30 × 27x44mm): the moderate hypo-echoic and irregular shaped PR (SOL) is located anterior and deep to the SLG and partially above and partially below the MM in the anterior part of the floor of the mouth and is reaching to the submandibular space (**a**, orange arrow). The low-flow doppler sonography shows that the PR is perfused also in the region of the plunging through the hiatus of the MM (diameter 23.3 mm, orange arrow, b). Video 1 of the supplemental material is corresponding to this imaging. Lateral transection view (**c**) with low-flow doppler sonography shows that the PR (SOL) is partially perfused, in particular in the region of the plunging through the hiatus of the MM (diameter 26.1 mm) and is extending to the submandibular space (orange arrow, C). A puncture of the SOL revealed secretion characteristic for ranula. To exclude any combined pathology (e.g. PR and vascular malformation), MRI was also indicated. Video 2 of the supplemental material is corresponding to this imaging. *GHM* geniohyoid muscle, *DM* digastric muscle, *MM* mylohyoid muscle, *SOL* space occupying lesion, *SLG* sublingual gland, *SMG* submandibular gland, *TONG* tongue, *M* mandible

**Fig. 4:**
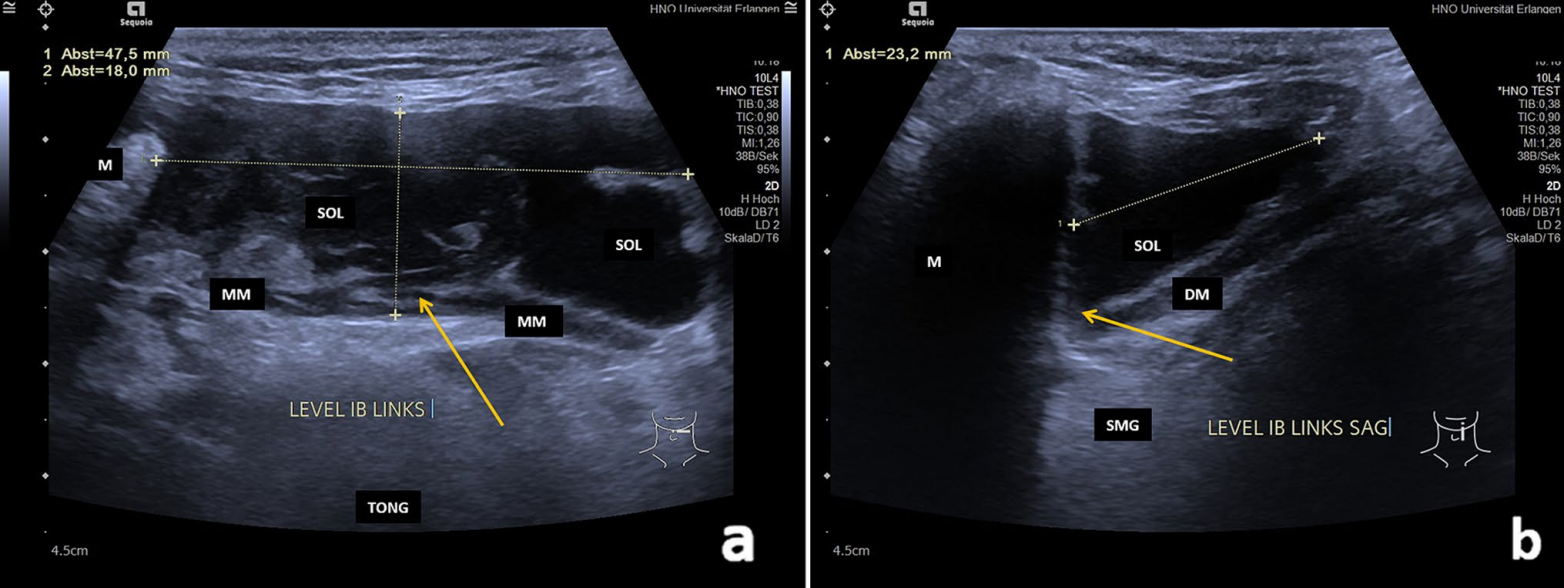
**a**–**b** Transection (**a**) and longitudinal view (**b**) of the floor of the mouth shows a PR (SOL) in the left floor of the mouth in a 69 year old male patient (size 48 × 18 × 23 mm): The SOL (PR) shows a hypo-echoic tissue texture and is irregular shaped. The anatomical structure of the MM shows no continuity due to a hiatus (orange arrow, A). The PR located deep to the SLG and there is nearly no contact to it. The PR (SOL) is located almost totally below the MM in the submandibular space nearby the SMG and below the niveau of the mandible (orange arrow, B). *DM* digastric muscle, *MM* mylohyoid muscle, *SOL* space occupying lesion, *SLG* sublingual gland, *SMG* submandibular gland, *M* mandible, *TONG* tongue, *Level IB links* Level IB left side

**Fig. 5:**
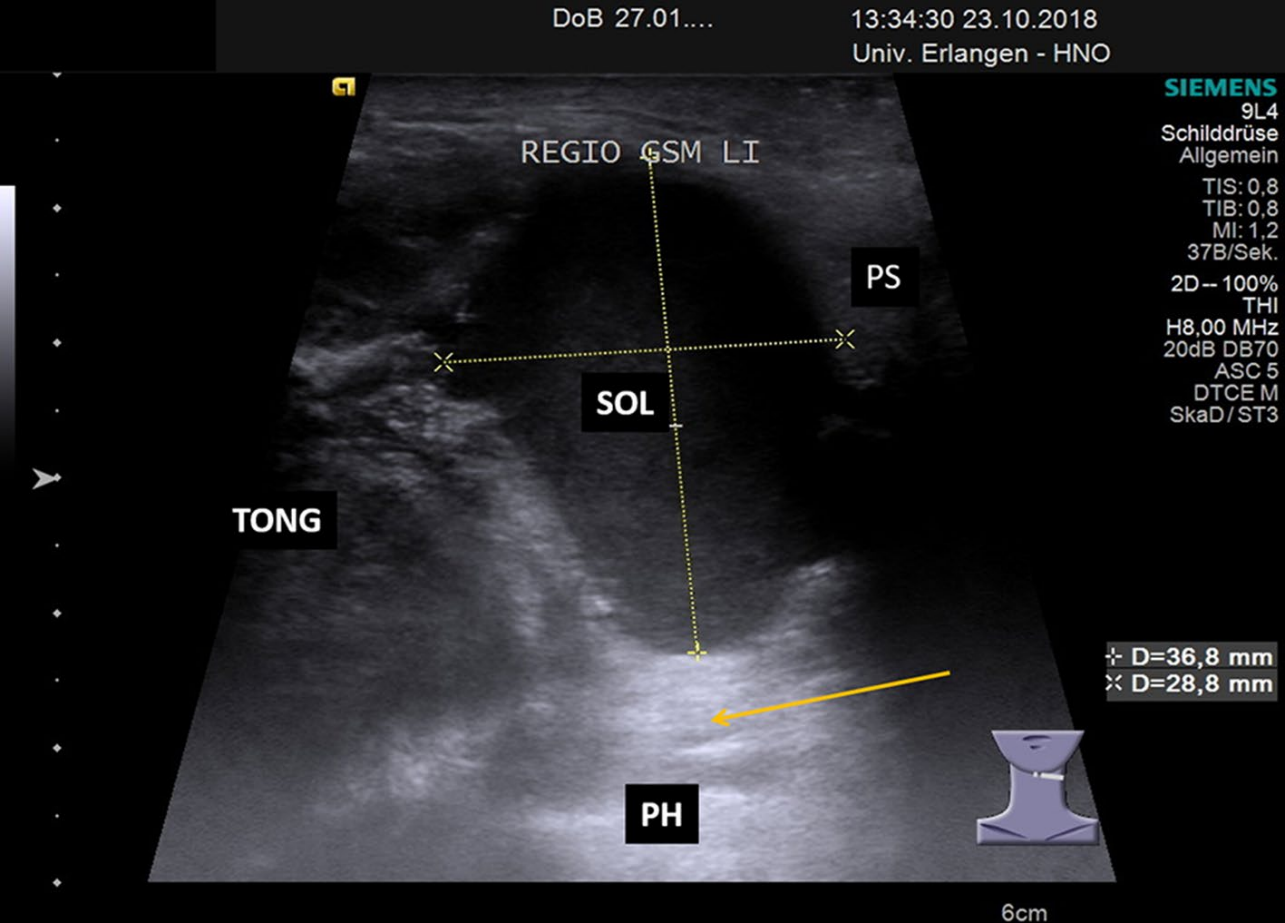
Transection view of the posterior submandibular space shows space occupying lesion dorsal to the tongue and the tonsil region in the transition to the PPS in a 34 year old male patient presenting with 2 recurrent ranulas on the left side (size of ranula located in the PS 37 × 18 × 28 mm). One PR (not depicted) was located primarily in the SS and the second in the PS. The hypo-echoic and regular shaped SOL is shown dorsal to the TONG and tonsil nearby the pharynx within the PS. The orange arrow indicates the reverberations effect caused by the air within the pharyngeal lumen. Video 3 of the supplemental material is corresponding to this imaging. *SOL* space occupying lesion, *TONG* tongue, *PH* pharynx, *PS* parapharyngeal space, *SS* submandibular space, *Li* left side

In all, 170 ERs were present in 169 patients (Fig. 1a–b). One patient had bilateral ERs. Thirty-seven PRs were diagnosed in 36 patients (Figs. 2–5). Two unilateral recurrent ranulas were present in one patient (Table 1). When ERs and PRs were compared, no differences between them were observed for age, gender, side, laterality, or for the echo texture, which was hypoechoic to anechoic in 97.6% of cases. Perfusion at or within the ranula was detectable with colour-coded Doppler ultrasound in two cases (both PRs; Fig. 3a–c, Table 1). These lesions were primarily suspicious for PRs, but puncture was performed and MRI was indicated in order to rule out any (accompanying) vascular malformation. PRs were significantly larger than ERs (43.75 ± 1.96 mm vs. 20.85 ± 0.98 mm; *P* = 0.0001). Only 20.59% of ERs were ≥ 30 mm in size (range 30–73 mm). PRs had an irregular shape significantly more often in comparison with ERs (94.6% vs. 10%; *P* = 0.0001).

**Table 1:** Data of ultrasound examination in all (n=207), ERs (n=170) and PRs (n=37)

Parameter	Ranulas total (n=207) number (%)	Enoral ranulas (n=170) number (%)	Plunging ranulas (n=37) number (%)	Exact-Test (Chi-Square or M-W U-Test): enoral vs. plunging ranulas
Age (years)	33.74 ± 1.2 (range 0.5–76, median 34.0)	34.24 ± 1.34 (range 0.5–76, median 35.0)	31.39 ± 2.69 (range 4-69, median 27.5)	n.s.
Gender (male vs. female)	107 (51.7%) vs. 98 (47.3%)	84 (49.4%) vs. 85 (50.6%)	23 (62.2%) vs. 13 (35.1%)	n.s.
Side (right vs. left)	93 (44.9%) vs. 114 (55.1%)	78 (45.6%) vs. 93 (54.4%)	15 (40.5%) vs. 22 (59.5%)	n.s.
Size maximum diameter	24.69±1.05 (M 22.0; range 4-73)	20.85±0.98 (M 17.0; range 4-73)	43.75±1.96 (M 44.0; range 13-68)	0.0001
Echo-free	202 (97.6%)	166 (97.6%)	36 (97.3%)	n.s.
Moderate hypo-echoic	5 (2.4%)	4 (2.4%)	1 (2.3%)	n.s.
Shape regular	154 (74.4%)	152 (89.4%)	2 (5.4%)	0.0001
Shape irregular	53 (25.6%)	18 (10.6%)	35 (94.6%)	0.0001
Perfusion	2 (0.97%)	-----	2 (5.41%)	-----
location partial over MM	27 (13.0%)	0 (0%)	27 (73.0%)	0.0001
location complete over MM	170 (82.12%)	170 (100%)	0 (0%)	0.0001
location partial below MM	29 (14.0%)	0 (0%)	29 (78.37%)	0.0001
location complete below MM	7 (3.4%)	0 (0%)	7 (18.9%)	0.0001
location over and below MM	28 (13.5%)	0 (0%)	28 (75.7%)	0.0001
prolapse of SLG through MM	6 (2.9%)	0 (0%)	6 (16.2%)	0.0001
location in relation to SLG: superficial	169 (81.6%)	158 (92.9%)	11 (29.7%)	0.0001
location in relation to SLG: deep	70 (33.8%)	34 (20.0%)	36 (97.3%)	0.0001
location in relation to SLG: anterior part	195 (94.2%)	168 (98.8%)	27 (73.0%)	0.0001
location in relation to SLG: posterior part	36 (17.4%)	25 (14.7%)	11 (29.7%)	n.s. (0.089)
location in the submandibular space	29 (14.0%)	0 (0%)	29 (78.37%)*	0.0001 ^+^
location in the parapharyngeal space	2 (1.0%)	0 (0%)	2 (5.4%)	0.030^+^
Plunging through a hiatus of MM	28 (13.5%)	0 (0%)	28 (77.8%)*	0.0001
Plunging posterior around the MM	7 (3,4%)	0 (0%)	7 (18.92%)*	0.0001

* 1 patient only submandibular space (recurrence at presentation after surgery alio loco)

+ One (recurrent) PR in SS and PS

In all cases, the ERs were located entirely above the level of the MM (Fig. 1a–b). By contrast, PRs were much more frequently located partly and/or completely below the level of the MM. ERs were located significantly more often superficially and anteriorly in relation to the SLG, whereas PRs were located deeper than the SLG significantly more often (*P* = 0.0001). There was a strong tendency for PRs to be located posterior to the SLG (*P* = 0.089). Signs of plunging and extension to the SS (*P* = 0.0001) or PS (*P* = 0.03; for details, see Table 1) were only observed in PRs, which was the basis for their diagnosis. Plunging beyond the posterior border of the MM was observed in 18.92% of PRs (Fig. 2a–b). Plunging of the ranula and/or SLG through a hiatus of the MM was observed in 77.8% of PRs. Defects in the MM were recognized using ultrasound in all cases in the anterior part (Fig. 3a–c, Supplementary Videos 1–2). Deeper extension beyond the level directly below the MM as far as the SS (Fig. 4a–b) or PS was recognized in 78.36% and 5.4% of PRs, respectively. Location in the PS was associated with recurrences in the two cases (Fig. 5, Supplementary Video.3). Location in the SS only below the MM was observed in 18.91%. A separate location in the PS only was noted in one patient.

## Discussion

Although ER can be easily diagnosed primarily by clinical examination, the precise size of larger ranulas that extend into deeper regions may not be adequately recognizable. Ultrasound can be used to define their exact extent. Various reports have described the use of ultrasound in ERs [1, 21–25] or PRs [1, 11, 15, 22–24, 26–28], but without detailed assessment. Closer descriptions of the ultrasound findings have appeared in case reports and small case series, particularly for pediatric patients [9, 18–20] and in reviews [1, 7, 10]. However, there have been no detailed analyses of the ultrasound features of ranulas that provide diagnostic criteria that can be used to distinguish between ER and PR.

Due to the content of ranulas, which consist of mucous saliva, they typically have a hypoechoic or even anechoic echo texture, with strong enhancement in the deeper regions (Figs. 1–5) [1, 6, 11, 17–20]. Sludge-like echoes may be present, and these appear to correlate with the thickness of the mucous secretion (Fig. 1a–b). Moderate echogenicity is very rarely present (2.4% of ERs, 2.3% of PRs in the present study). More intensive echoes may be caused by cicatrization and/or by additional pathologic changes, such as hemorrhagic transformation [29]. In addition to the echogenicity of ranulas, their location, their relationship to neighboring anatomical structures such as the MM, the plunging pattern, and perfusion are of paramount importance for the diagnosis and differential diagnosis. The differential diagnosis includes lymphangioma, epidermal cysts of the floor of the mouth, branchial cysts with a median location, and other benign space-occupying lesions, and it may sometimes be difficult [10, 12, 14–16, 30–32]. Lymphangiomas, branchial cysts or cervical cysts typically have an echo-poor internal tissue texture, which complicate its differentiation. Lymphangiomas and cystic hygromas frequently show pronounced septation (which is minimal or absent in ER/PR) and no signs of plunging. By contrast, epidermal cysts, lipomas, and ectopic thyroid tissue tend to have a denser (hyperechoic or mixed) tissue texture, do not have an anatomical relationship to the sublingual gland or MM, and are found in more median, caudal, or lateral locations [30]. PRs may have a submandibular, parapharyngeal, or other unusual location. The differential diagnosis between PRs and other space-occupying lesions in the SS and PS can sometimes be very difficult. The occurrence of primary ranulas solely in the SS or PS^14^, ^31^, ^33^, ^34^ or even in the thoracic space [35] was described in very few reports. In the patients included in the present study, 78.37% were located in the SS, but  18.91% of PRs were observed  in the SS only and 5.4% in the PS. For the differential diagnosis, further diagnostic measures such as MRI or diagnostic puncture of the lesion may be helpful to confirm the suspected diagnosis in such cases [15]. Perfusion is typically absent in ranulas, as their wall consist of saliva surrounded by a thin granulomatous membrane [1, 16]. Increased perfusion was noted in 5.4% of PRs, predominantly in the periphery or in the region of the hiatus of the MM, where the plunging occurred (Fig. 3a–c). In these cases, MRI and diagnostic puncture can also be used to rule out any vascular malformation or combined pathology and prepare the subsequent treatment appropriately [15].

Ultrasound does not present any difficulties in smaller ERs, but can be very helpful in larger ones (≥ 30 mm) to exclude PR [1, 21–25] In the present study, 20.59% of ERs had a maximum diameter of ≥ 30 mm (range 30–73 mm). The pattern of spread and extension of ERs and PRs can be used both for differential diagnosis in general and also to distinguish between the two, which is important for further planning of their management, as the therapeutic approaches may differ considerably [1, 6, 11, 17–20]. Several clear ultrasound criteria were identified for differentiating between the two types. PRs significantly more often were larger and had an irregular shape. PRs were located significantly more often partly above and below or completely below the level of the MM, but in no cases completely above it. By contrast, all ERs were completely located above the MM. PRs were also located superficial to the SLG and anterior to it significantly less often, and deep to the SLG significantly more often in comparison with ERs. PRs showed a strong tendency to be located posterior to the SLG more often in comparison with ERs. Plunging through a hiatus of the MM, extension over the posterior border of the MM, prolapse of the SLG through a MM hiatus, and extension into the SS or PS were only observed in PRs (and in all of them) and represented characteristic ultrasound signs. Perfusion was observed only in PRs, typically within a hiatus in the MM (Table 1).

Ultrasound is more complex in plunging ranulas, and several studies performed by one research group deserve a closer look. A thorough investigation of diagnostic aspects of PRs was published recently by Jain et al. in several reports highlighting the role of ultrasound in these lesions [6, 11, 12, 17]. The studies provide a detailed analysis of various aspects of PRs and information useful for their management. One study reported that 98% of PRs were associated with plunging through a MM hiatus [17], comparable to the 77.8% rate seen in the present study. Another study evaluated differences in the herniation of the SLG through a MM hiatus [17]. In contrast to the results published there (prolaps of SLG observed in nearly all cases), a spontaneous and unprovoked prolapse of the SLG was visible in only 16.2% in the present study (Fig. 3b, Supplementary Video.2). However, when the intraoral pressure was increased, a temporary prolapse of the SLG was observable also in nearly all cases. If herniation was recognizable, a “slide-type herniation” was most often observed. It has to be mentioned in that context that cases with herniation of the SLG without manifest PRs were not included in the present analysis. In an additional study, a “tail sign” was described and was observed in 1.6% of 126 consecutive PR cases [6]. It was attributed to a small and longitudinal hypoechoic band located above the level of the MM in PRs that extended beyond the posterior border of the MM. In the present study, extension of ranulas up to the posterior region above the MM was observed in 29.7% of the PRs, but also in 14.7% of the ERs, with no significant differences observable between the different types of ranula (*P* = 0.89, Table 1). If identical criteria concerning the extension pattern are included, a pattern very similar to the “tail sign” was not recognizable in the present cases.  However an  extension somewhat similar to the “tail sign” was noted in 18.92% of PRs in the present study, in comparison with the 1.6% reported by Jain et al. The hypoechoic band between the MM and the SLG was much broader or even oval or hourglass-shaped in the present PR cases (Fig. 2a–b). However, like Jain et al., we would also conclude that absence of a typical tail sign “does not exclude the diagnosis of a plunging ranula.” [6].

Overall, on the basis of the present results and those in the literature, it can be concluded that a hypoechoic space-occupying lesion has a high likelihood of being a PR if it has an irregular shape and a distinct relationship to the MM (partly above and below or completely below its level) or the SLG (with extension into the space deep and posterior to the SLG) and extension into the SS or PS. A hypoechoic space-occupying lesion with a regular shape that is located above the MM, with no hiatus visible, strongly suggests the presence of an ER. In larger lesions, signs of plunging (lesion and/or SLG) must be evaluated carefully. If perfusion is visible (through a hiatus or in the lesion itself), the presence of a vascular malformation must be at least considered, particularly a vascular malformation and/or lymphangioma or hygroma.

One weakness of this analysis is its retrospective nature and the design. We are aware of some bias in the investigation, which was primarily focused on identifying and describing ultrasound findings to define the diagnosis of ERs and PRs and distinguish between them. The statistical analysis was adequate for parameters that are not dependent on ultrasound findings such as the patient’s age and gender or the size, shape, and location of the lesion in relation to the SLG. A few parameters describing the location of ranulas relative to the MM or plunging patterns were crucial for defining the diagnosis. Ultrasound-based findings and diagnoses themselves were therefore not independent. Nevertheless, statistics were used for these parameters in order to illustrate differences between ERs and PRs on the basis of ultrasound findings, with highly significant differences between ERs and PRs (Table 1). Our experience suggests that incidental ERs or PRs are very unlikely, or can even be excluded, if there are no clinical manifestations or symptoms and a space-occupying lesion is not visible intraorally or in the submandibular region. There is a possibility that very small and deep located ranulas may be overlooked primarily as these may cause no clinical complaints. However, as ranula tend to grow in nearly all cases, also such rare cases will cause symptoms over the course of time and will then be diagnosed by clinical investigation followed by ultrasound examination as the most probable primary imaging method.

The sonographic criteria elaborated in this manuscript and the results published in the literature underscore that ultrasound is an excellent diagnostic tool for diagnosing ranula and differentiating between ER and PR. Ultrasound can be repeated as often as needed, is not associated with exposure to ionizing radiation as in CT scanning, and is a fast and inexpensive investigation method in comparison with CT and MRI. In addition, video storage of the ultrasound findings can be helpful for repeated and detailed analysis. Ultrasound also allows appropriate planning of subsequent surgical treatment, which is particularly important in patients with PR and/or younger patients [1, 6, 11, 17–20].

## Conclusions

The published results underline the value of ultrasound as a first-line diagnostic tool to allow detailed diagnosis of ranulas and distinguish between ERs and PRs. In addition, ultrasound performed by the surgeon can be very useful for preoperative and intraoperative purposes. MRI or ultrasound-guided puncture should be reserved for assessing the extent and ramifications of very large and/or recurrent plunging ranulas, or in space-occupying lesions with an unclear echo texture and signs of perfusion [9, 13, 15].

## Abbreviations

ER: Enoral ranula
PR: Plunging ranula
US: Ultrasound
DD: Differential diagnosis
MM: Mylohyoid muscle
SLG: Sublingual gland
SS: Submandibular space
PS: Parapharyngeal space
